# MiR-503 Promotes Bone Formation in Distraction Osteogenesis through Suppressing Smurf1 Expression

**DOI:** 10.1038/s41598-017-00466-4

**Published:** 2017-03-24

**Authors:** Yuxin Sun, Jia Xu, Liangliang Xu, Jinfang Zhang, Kaiming Chan, Xiaohua Pan, Gang Li

**Affiliations:** 1 Department of Orthopaedics and Traumatology, Bao-An District People’s Hospital, Shenzhen, P.R. China; 2 Department of Orthopaedics & Traumatology, Li Ka Shing Institute of Health Sciences and Lui Che Woo Institute of Innovative Medicine, Faculty of Medicine, The Chinese University of Hong Kong, Prince of Wales Hospital, Shatin, Hong Kong SAR P.R. China; 3 The CUHK-ACC Space Medicine Centre on Health Maintenance of Musculoskeletal System, The Chinese University of Hong Kong Shenzhen Research Institute, Shenzhen, P.R. China; 4 Department of Orthopaedic Surgery, Shanghai Jiaotong University Affiliated Sixth People’s Hospital, Shanghai, P.R. China; 5 Key Laboratory for Regenerative Medicine, Ministry of Education, School of Biomedical Sciences, Faculty of Medicine, The Chinese University of Hong Kong, Hong Kong SAR, P.R. China

## Abstract

Distraction osteogenesis (DO) is a unique technique for promoting bone formation in clinical practice. However the underlying mechanism remains elusive. As epigenetic mediators, microRNAs have been reported to play important roles in regulating osteogenesis. In this study, after successfully established the DO model of rats, a microRNA microarray was performed to find molecular targets for DO. Total 100 microRNAs were identified as differently expressed, with miR-503 being one of the most significantly up-regulated miRNAs in DO. The further investigation also showed that miR-503 was upregulated during osteogenesis in mesenchymal stem cells of rats, and overexpression of miR-503 significantly promoted osteogenesis *in vitro* and accelerated mineralization in DO process *in vivo*. By using bioinformatic investigations and luciferase activities, we successfully demonstrated that Smurf1, a negative regulator of osteogenesis, was a real target of miR-503. Furthermore, Smurf1 knockdown promoted osteogenesis and antagomir-503 abolished the promotive effect, suggesting that miR-503 mediated osteogenic differentiation via suppressing Smurf1 expression. To sum up, these findings indicated that miR-503 promoted osteogenesis and accelerated bone formation, which may shed light on the development for a potential therapeutic target for bone repair.

## Introduction

Distraction osteogenesis (DO) is a unique technique for bone regeneration which has been widely applied in limb lengthening and reconstruction. Regular tensile forces are performed across the osteotomy gap guided by the external fixation in DO process so that the large volumes of new bone could be developed. The process of DO could be divided into three different phases, including latency, distraction and consolidation phase. Initially, bone segments are strictly fixed for 5–7 days after the osteotomy. Then controlled distraction is performed until the desired lengthening is obtained. Finally, the external fixation is maintained in place without distraction until the newly formed bone is mechanically strong enough so that the external fixation could be safely removed.

Several biological factors have been reported to be involved in the regulation of bone regeneration in each phase of DO process. For example, some inflammatory cytokines, such as IL-1 and IL-6^1^, are released from osteotomy area to recruit stem cells and initiate the repair cascade in the early latency phase. Some growth factors, such as bone morphogenetic proteins (BMP)^2^, transforming growth factor β (TGF-β)^3^, fibroblast growth factors (FGF)^4^, epidermal growth factor (EGF)^5^ are upregulated during the distraction phase and tapered off during the consolidation phase. Therefore, the upregulation of growth factors would also raise the maximal biological response of distracted area to bone regeneration. Besides, more regenerated bone would be created in DO procedure than in simple fracture healing process^6^. It has been proved that some key factors, such as inducible nitric oxide synthase^7^, vascular endothelial growth factor (VEGF), tissue inhibitors of metalloproteinase-1 (TIMP-1)^8^ and stromal cell-derived factor-1 (SDF-1)^9^, were more released in distraction gap during DO process, contributing to the rapid bone regeneration. Although these biological factors only partially explain the reason of bone regeneration in DO, the detailed molecular mechanisms of DO in bone formation are still not well understood.

Recently, lots of studies have indicated that miRNAs play important roles in skeletal development and homeostasis. MicroRNAs belong to noncoding small RNAs family, 21–25 nt in length, encoded in the genome, which can regulate the gene expression by targeting 3′ untranslated region (UTR) of mRNAs at posttranscriptional level^10^. It has been speculated that more than 60% human protein coding genes could be regulated by microRNAs^11^. Several pivotal microRNAs were found to regulate the osteogenic differentiation *in vitro* and fracture healing process *in vivo*. For example, miR-17^12^ and miR-100^13^ were up regulated while miR-10a^14^ and miR-205^15^ were downregulated during the osteogenic differentiation in bone mesenchymal stem cells of rats (rBMSCs). Overexpression of miR-125b^16^ would inhibit osteogenic differentiation while anti-miR-221^17^ intervention would trigger the osteogenic differentiation. Besides, miR-140, miR-181a-5p and miR-451 expression were all highly increased during fracture healing process^18^ and miR-21 overexpression^19^ or miR-92a knockdown^20^ would enhance fracture healing property. Combined all these studies, we hypothesized that some crucial microRNAs could regulate the process of distracted bone healing, which could be used as therapeutic targets to promote bone formation.

In this study, we performed a comparative microRNA profiling to identify the differently expressed miRNAs and miR-503-5p (miR-503) was found to be the promising candidate in DO animal model. The further investigation also showed that miR-503 triggered osteogenesis *in vitro* and promoted bone formation *in vivo* through suppressing Smurf1 expression, which serves as an inhibitor of osteoblast. These findings provide insights for developing a new therapeutic strategy to bone regeneration.

## Results

### MiR-503 expression is increased during the distraction period of DO and osteogenic differentiation of rBMSCs

**Figure 1:**
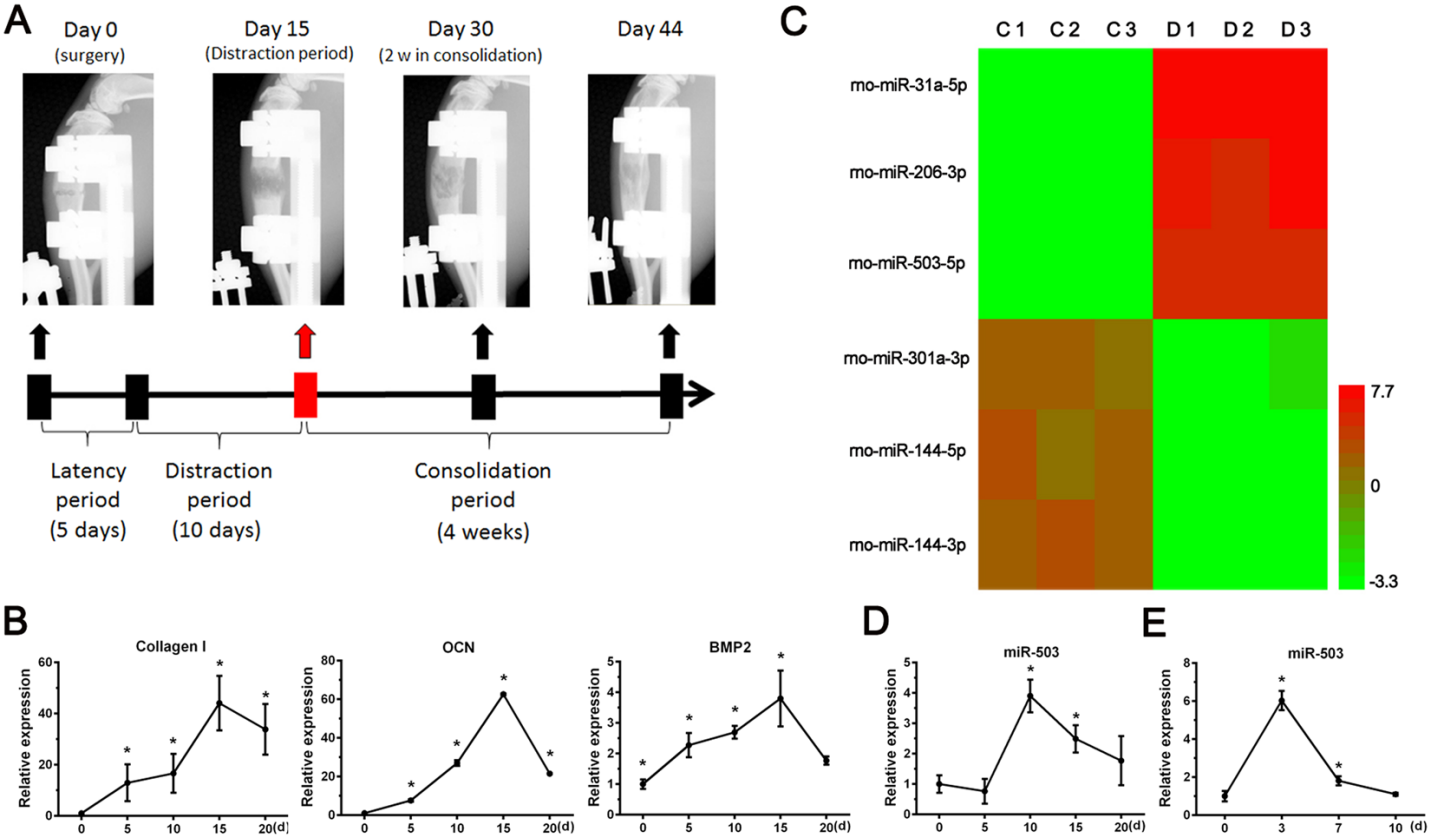
MiR-503 expression is increased during DO process and osteogenic differentiation. (**A**) Animal model of DO in rats. Bone formation was obvious in the distraction gap during distraction phase detected by X-ray. At the end of distraction phase, bone tissue from distraction gap were harvested for a microRNA microarray test. The contralateral tibiae were also harvested for normal control (n = 3 per group). (**B**) Expression level of Collagen I, OCN and BMP2 were detected by q-PCR with the bone samples harvested from distraction gap at different observation time points. Results showed that these osteogenic markers were increased in different degrees during the DO process (n = 3 per group, each time points, *P < 0.05 compared to Day 0, GAPDH was used as an internal reference). (**C**) Heatmap of microarray result. (Simplified version). The top three up/down regulated microRNAs were list in the result. Red color represents up regulation while green color represents down regulation. C represents control group, D represents distraction group (n = 3 each group). (**D**) MiR-503 expression during DO process. Animals received DO surgery were sacrificed at day 0, 5, 10, 15 and 20. Fold change of miR-503 expression level at different time points were calculated through compared to the expression level at day 0. (n = 3 each time points, *P < 0.05 compared to Day 0, U6 was used as an internal reference). (**E**) MiR-503 expression in rBMSCs during osteogenic differentiation. Osteogenic induction was performed in rBMSCs. Cells were harvested at day 0, 3, 7 and 10. Fold change of miR-503 expression at different time points were calculated through compared to the expression level at day 0. (n = 3 each time points, *P < 0.05 compared to Day 0).

After successfully established the DO model of rats (Fig. 1A), bone tissues of distraction gap were harvested at different observation time points. Expression level of osteogenic markes, such as Collagen I, OCN and BMP2, were detected by qPCR. The result showed these markers were increased in different degrees during the DO process. All markers could be found highly expressed at the end of distraction phase (Day 15) (Fig. 1B). Therefore, a miRNA microarray assay was performed with the bone samples harvested at day 15. According to the dramatic changes showed in the heat-map results, miR-503 is of great interest due to its one of the most upregulation in all the samples (Fig. 1C). To verify the results of microarray, we also took advantage of qPCR and the results confirmed that miR-503 was significantly upregulated at the distraction period of DO and early stage of osteogenesis of bone mesenchymal stem cells of rats (rBMSCs) (Fig. 1D). These data indicated that the elevated expression of miR-503 would play important roles in regulating calcification in DO and osteogenic differentiation of rBMSCs.

### MiR-503 overexpression promotes osteogenic differentiation of rBMSCs

**Figure 2:**
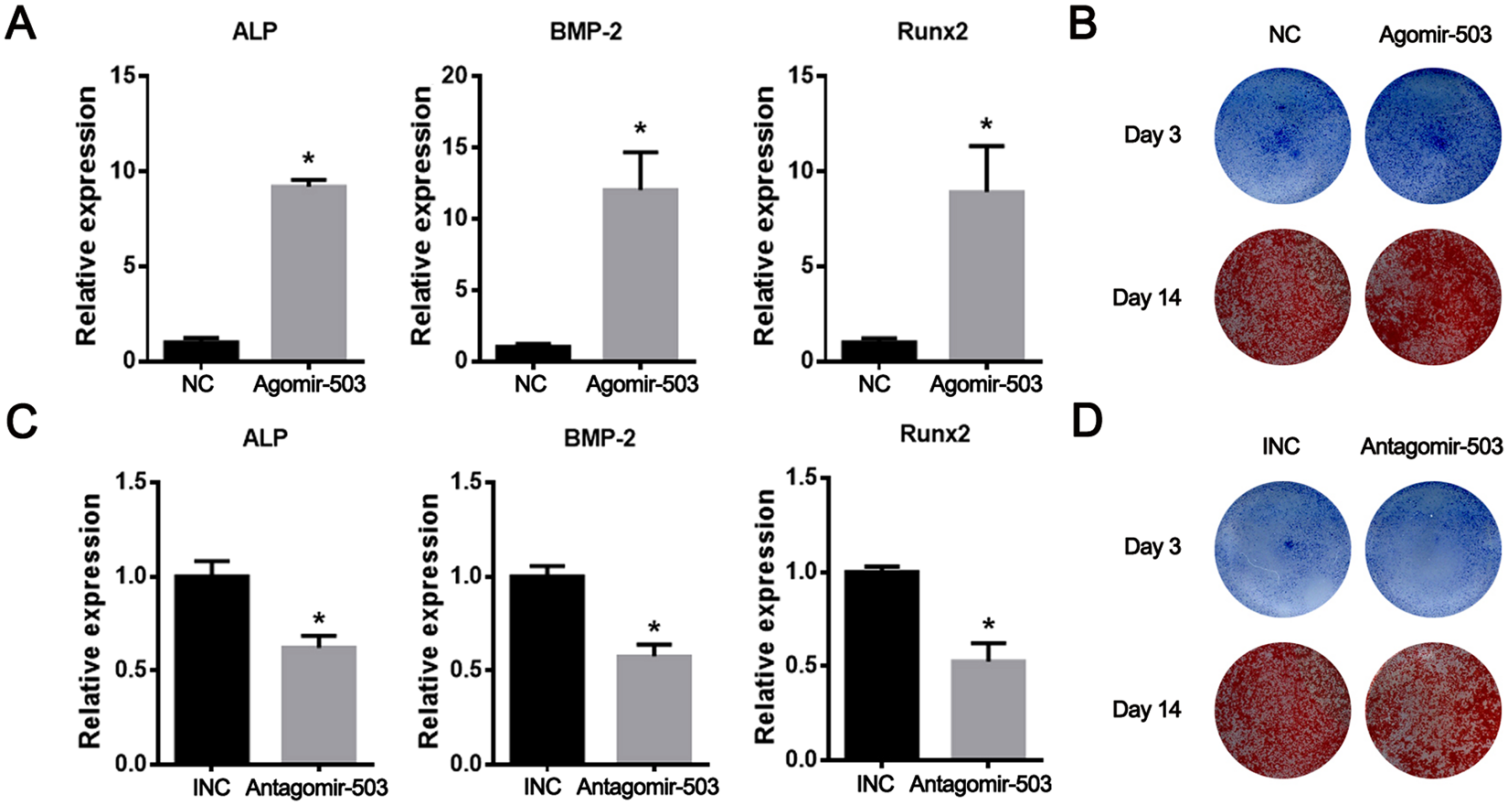
MiR-503 overexpression promotes osteogenic differentiation of rBMSCs. (**A,B**) Agomir-503 and NC were transfected into the rBMSCs by lipofectamine 2000. Three days after transfection, osteogenic differentiation was performed. Expression levels of ALP, BMP2 and Runx2 were detected by q-PCR three days after osteogenic differentiation. ALP and Alizarin red staining were performed 3 days and 14 days after the osteogenic differentiation. The result showed the osteogenic capacity of rBMSCs was significantly promoted by miR-503 overexpression. (**C,D**) As shown in the result, osteogenic capacity of rBMSCs was suppressed after antagomir-503 transfection. (n = 3 each group, *P < 0.05).

To elucidate the biological effect of miR-503 on osteogenesis of rBMSCs, agomir-503 and antagomir-503 were transiently transfected into rBMSCs and the expression of several osteogenic marker genes were examined. As shown in Fig. 2A, agomir-503 activated the expression of osteogenic markers such as ALP, BMP-2 and RUNX2. ALP and Alizarin red staining results also showed that osteogenic capacity of rBMSCs was enhanced after agomir-503 transfection (Fig. 2B). On the contrary, antagomir-503 intervention significantly suppressed these marker genes expression (Fig. 2C). ALP positive cells and calcium nodules were less detected in the staining result after antagomir-503 transfection (Fig. 2D). These results suggest that overexpression of miR-503 could promote osteogenic differentiation in rBMSCs.

### Smurf1 is a bona fide target of miR-503

**Figure 3:**
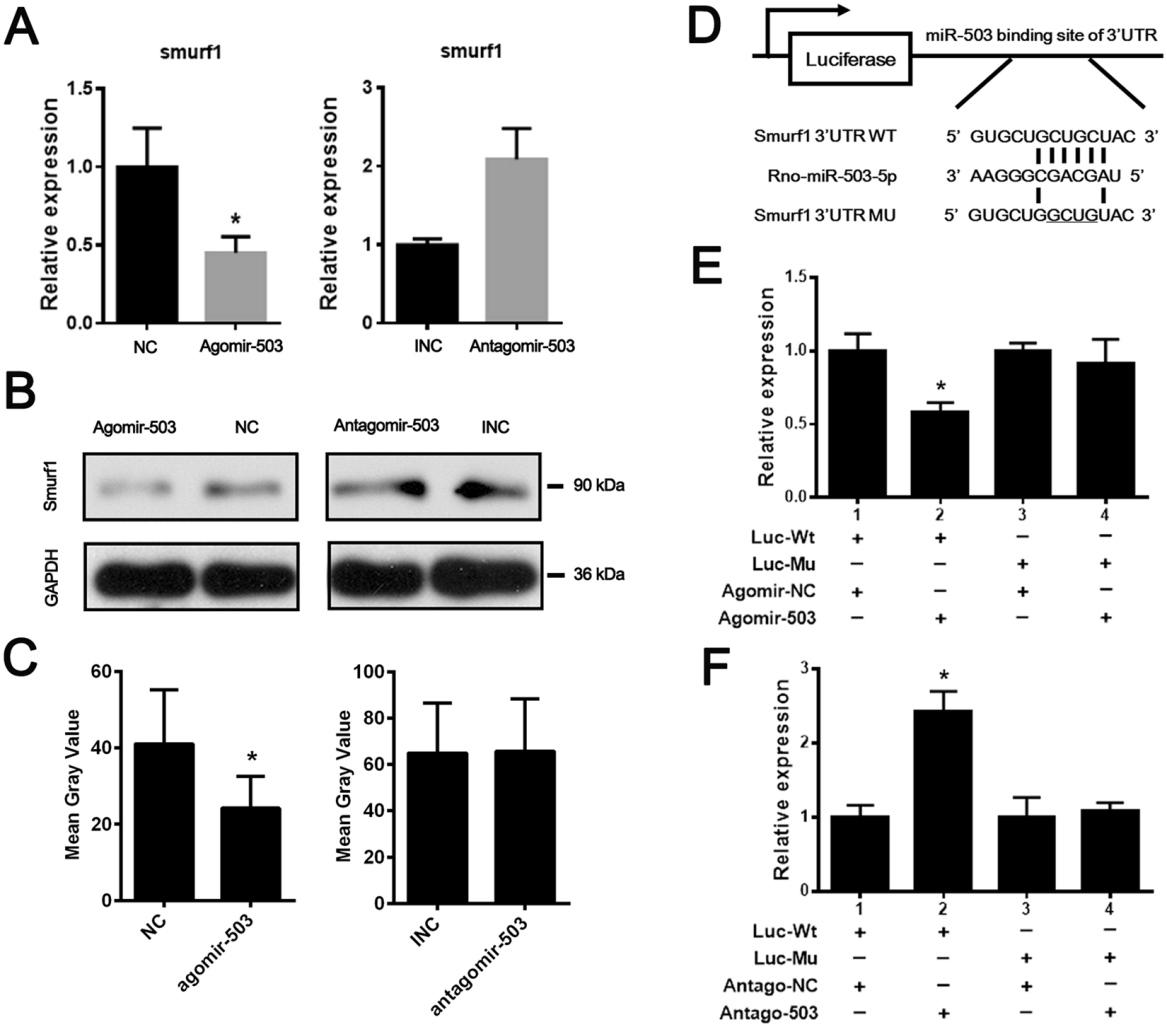
MiR-503 directly targets Smurf1. (**A**) Smurf1 expression was significantly decreased by agomir-503 at mRNA level. (n = 3, *P < 0.05). (**B**) Smurf1 protein expression was decreased by agomir-503 transfection while increased by antagomir-503 at protein level. (**C**) The result of western blot bends was quantified by the mean gray value. Significant difference in gray value could be found after agomir-503 transfected, compared to the NC group. However, no difference could be found after antagomir-503 transfected. (**D**) Schematic diagrams of the luciferase reporter construction. Wild type (Wt) and mutant (Mu) 3′ UTR of Smurf1 containing the binding site with miR-503 were inserted into pMIR-GLO vector. (**E,F**) HEK293 cells were transfected with miRNA oligoes combined with luciferase reporter (Wt). The effect of miR-503 on the luciferase activity was measured by luciferase reporter assays. MiR-503 could suppress while its inhibitor promote the luciferase activity. On the other hand, the miR-503 binding sites were mutated and the mutated luciferase reporters were co-transfected with agomir-503 or antagomir-503. The mutations on binding sites abolished the previously suppressive or promoted effects. (n = 3 each group, *P < 0.05).

It is well known that miRNAs exert their function through suppressing the target gene expression. Among the candidates predicted by the bioinformatics analysis, we found that Smurf1 is of great interest, in which its 3′-untranslated region (UTR) was predicted as a potential binding site of miR-503. By transfecting rBMSCs with miRNA mimics, we demonstrated both miR-503 significantly downregulated the mRNA and protein levels of Smurf1 (Fig. 3A–C). In order to validate whether it is a bona fide target for miR-503, we inserted the target sites into the 3′ UTR locus of firefly luciferase (Fig. 3D). It was showed that miR-503 dramatically suppressed the luciferase activity when compared with control groups and mutations on the binding sites successfully abolished the suppressive effects (Fig. 3E,F). Taken together, our results demonstrated that Smurf1 was a real target of miR-503 in rBMSCs.

### Smurf1 involves in the miR-503 mediated osteogenesis

**Figure 4:**
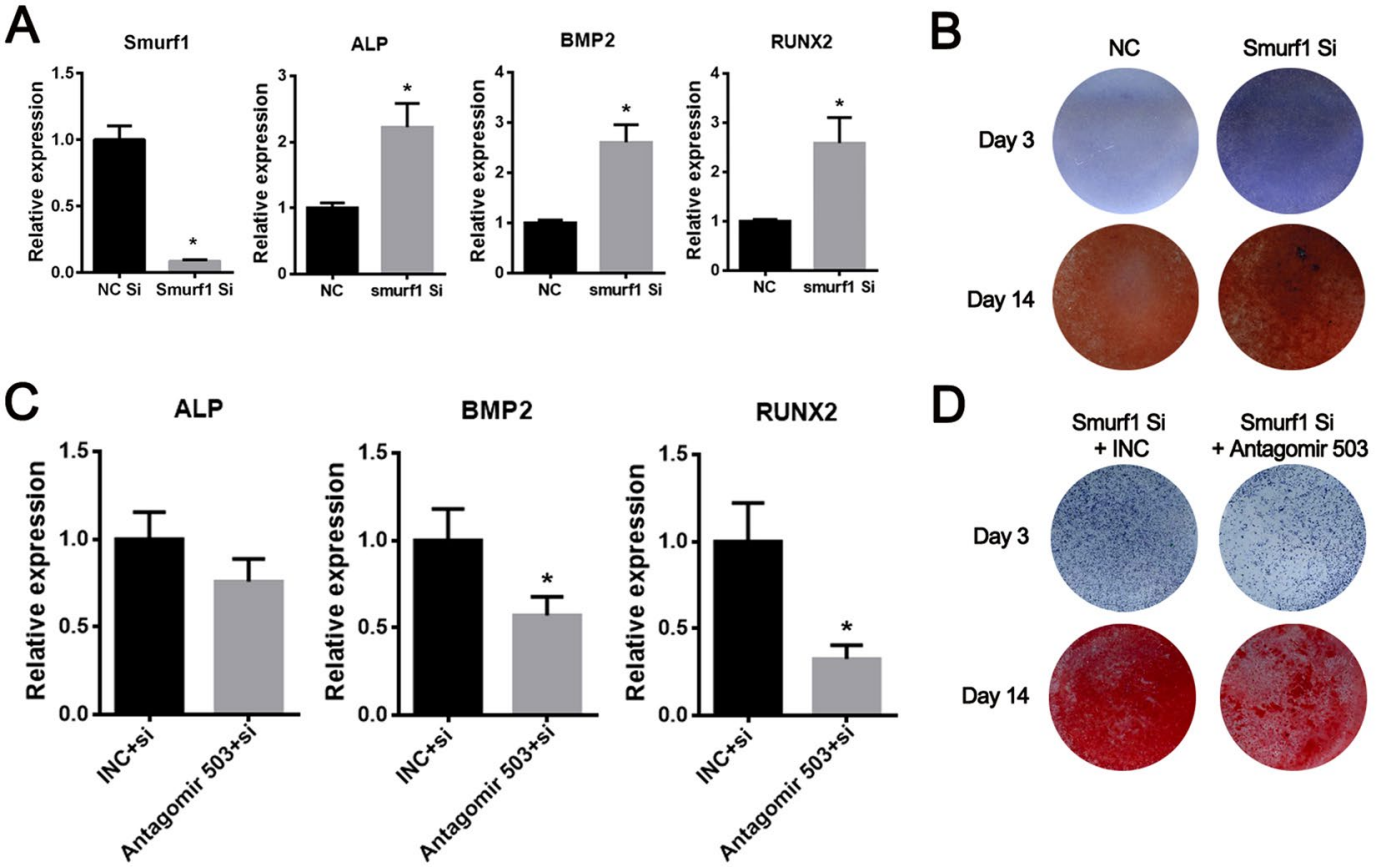
Smurf1 involves in miR-503 induced osteogenic differentiation in rBMSCs. (**A**) Expression level of smurf1 was significant down regulated after smurf1 siRNA transfection. Consequently, expression levels of ALP, BMP2 and Runx2 were increased by Smurf1 knockdown. (**B**) ALP and alizarin red staining were performed 3 days after Smurf1 siRNA transfection under osteogenic induction. Result showed that more ALP positive nodes and calcium nodes were observed in Smurf1 knockdown group. (**C,D**) The antagomir-503 reversed the upregulation of BMP2 and Runx2 induced by Smurf1 siRNA and it also reduced the promoted effect of Smurf1 knockdown on ALP & Alizarin Red staining. (n = 3, *P < 0.05).

As we all know, Smurf1 has been reported to be a negative regulator in osteogenesis. To elucidate whether the osteogenic effect of miR-503 was mediated by the suppression of Smurf1 in rBMSCs, a small interfering RNA (siRNA) specifically targeting Smurf1 was designed and its osteogenic effect was monitored. It was showed that Smurf1 knockdown significantly promoted the expression levels of ALP, BMP-2 and Runx2 (Fig. 4A). The further ALP and Alizarin red staining results also confirmed the promoted osteogenic effect induced by Smurf1 knockdown (Fig. 4B). Furthermore, we also observed that miR-503 inhibitor significantly abrogated the upregulation of osteogenic markers (Fig. 4C) and sensitized the promoted osteogenic effect (Fig. 4D) induced by Smurf1 knockdown. Collectively, these data demonstrate that Smurf1 involves in the miR-503 mediated osteogenesis.

### Local injection of miR-503 overexpressing rBMSCs enhances bone formation in DO animal model

**Figure 5:**
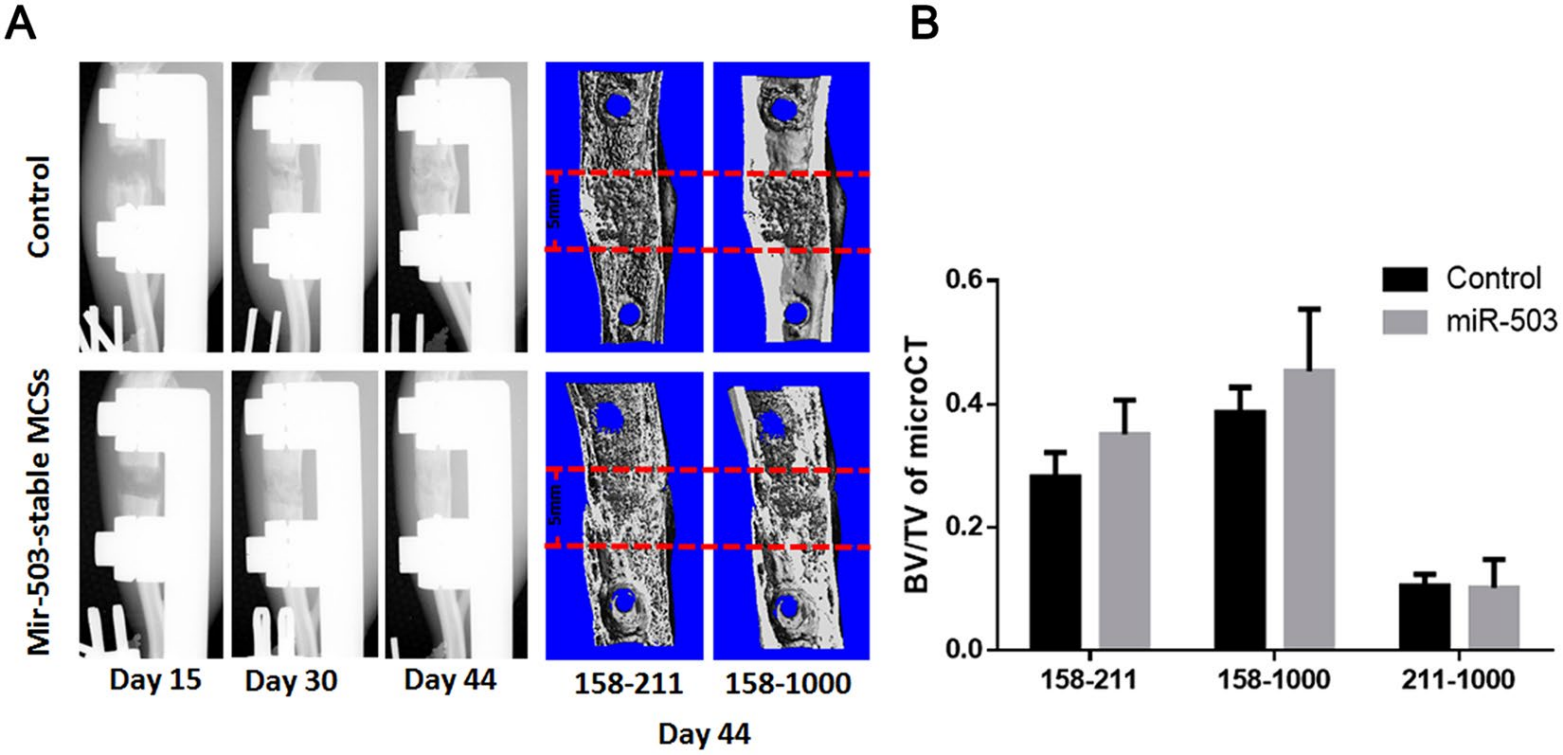
X-ray and microCT analysis confirm miR-503 overexpression therapy promote bone formation. (**A**) X-ray was examined at day 15, 30 and 44. Series of representative radiography were shown. The structure of the distracted bone had already repaired as normal at day 44 postoperation in miR-503 intervention group. A large amount of calluses around the distraction gap still could be found in control group. At 44 days postoperation, the central 150 layers bone tissues within the distraction gap were analyzed by microCT examination. (Vertical interval between red dotted lines represented the distraction gap). (**B**) Using microCT Quantitative analysis, result showed BV/TV was higher in miR-503 group between the thresholds 158 and 211. (n = 10 each group, *P < 0.05).

**Figure 6:**
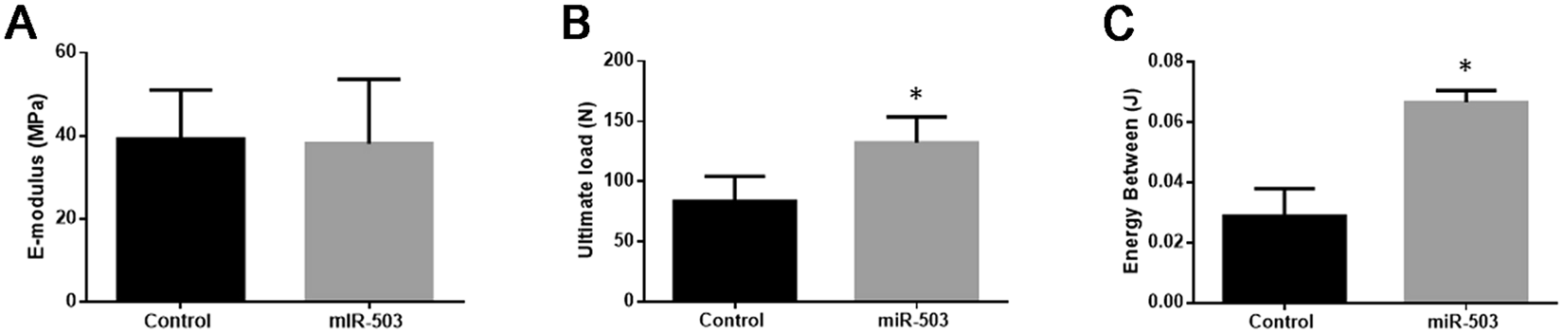
Bone healing property is enhanced by miR-503 overexpression therapy. Mechanical test was performed on the lengthened tibia after all the animals sacrificed at day 44 postoperation. Form the results, no significant differences was found in E-modulus (**A**) between these two groups. However, ultimate load (**B**) and energy between (**C**) were much higher in miR-503 group than that in control group (n = 10 each group, *P < 0.05).

**Figure 7:**
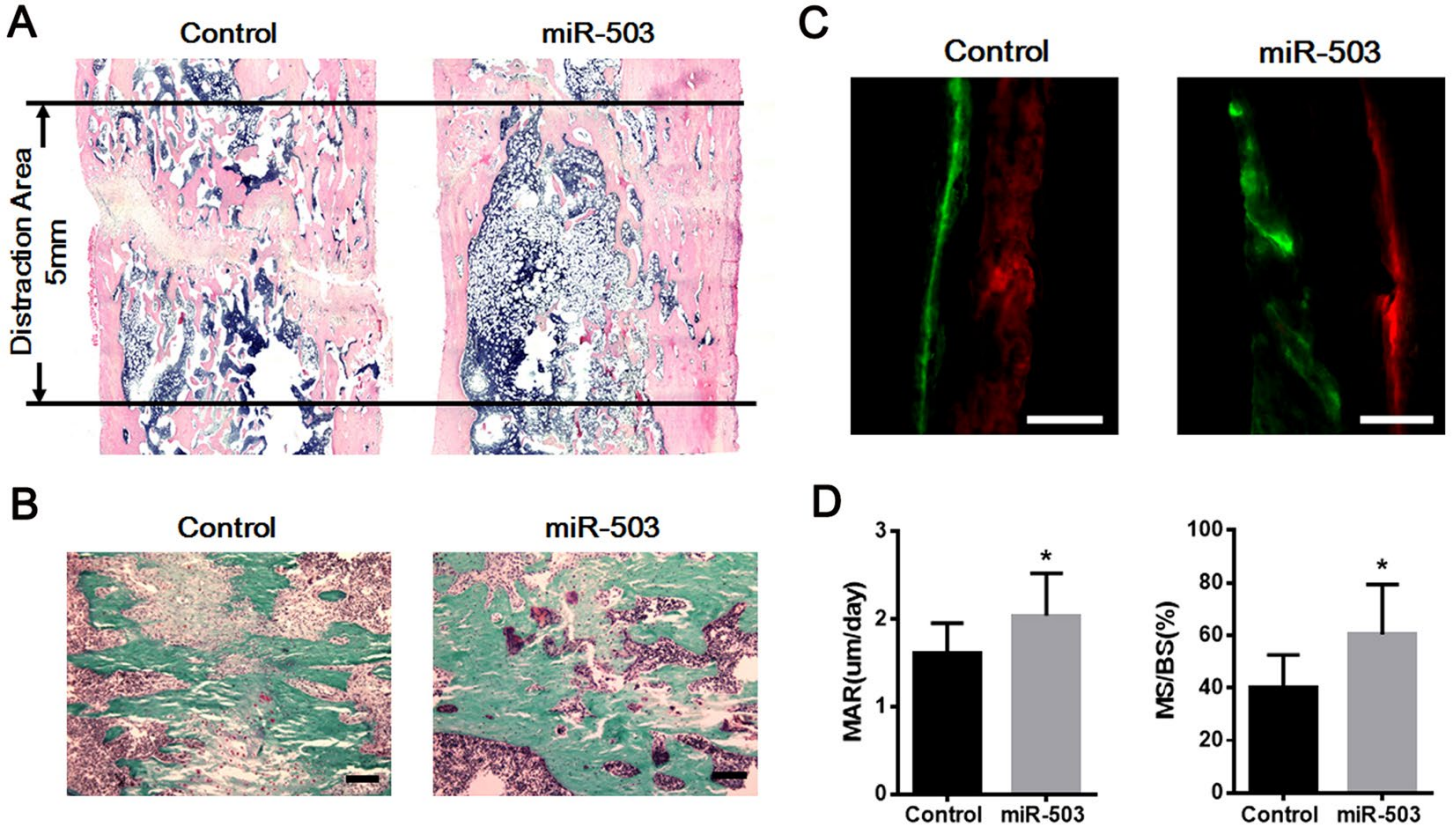
Bone tissue regeneration is accelerated by overexpression miR-503 therapy in DO animal model. (**A**) From HE staining of lengthened tibia in sagittal section, most of the regenerated bone tissues were mature in distraction gap in miR-503 overexpression group, while lots of cartilaginous callus still could be found in distracted gap in control group. (**B**) From Goldner’s trichrome staining, result showed that bone remodeling was vigorous in miR-503 overexpression group while lots of cartilage cells and fiber tissues still could be found in the distraction area. (Scale Bar = 150 μm). (**C**) Calcein (green) and Xylenol (orange) were subcutaneously injected into the rats at day 15 and day 43 separately. Vertical interval of the lines represented the amount of newly formed bone between the injection interphase. Result indicated miR-503 overexpression could promote mineralization of newly formed bone. (Scale Bar = 100 μm). (**D**) The dynamic histomorphometric parameters including MAR and MS/BS were found higher in miR-503 overexpression group than that in control group. (n = 5 each group, *P < 0.05).

To test the *in vivo* function of miR-503 in DO process, miR-503 overexpressing rBMSCs were developed and locally injected into the distraction gap at the end of distraction period. Eight weeks after injection, higher bone volume fraction (BV/TV) was observed in distraction gap in miR-503 overexpression group by microCT examination (Fig. 5A,B).Tibiae of the group with miR-503 overexpressing rBMSCs also showed better mechanical properties than the control group in ultimate load and energy by mechanical testing (Fig. 6). Histological analysis indicated that bone regeneration and remodeling were vigorous in miR-503 group while still lots of fiber tissue remained in the distraction area in control group (Fig. 7A). Besides, the regenerated bone was much more mature in distraction gap in miR-503 overexpression group than that in control group (Fig. 7B). The mineral apposition rate (MAR) and mineral surface versus bone surface (MS/BS) results also indicated bone regeneration was accelerated due to miR-503 intervention (Fig. 7C,D).

## Discussion

Large amounts of bone could be regenerated by DO technique, however the underlying mechanism of DO has still not yet completely revealed. Numerous of studies have tried to explain the mechanism from the point of mechanical stimulation and bone related growth factors, but regulatory function of microRNA in DO has never been mentioned before. In this study, miR-503 was screened from microRNA array and we also identified that it promoted osteogenic differentiation in rBMSCs and enhanced bone regeneration in DO animal model through suppressing Smurf1 expression.

MiRNAs function as gene silencers and are emerging as important regulators of gene expression and biological processes. Considering that multiple miRNAs have been demonstrated to play important roles in osteogenesis and bong formation, it is necessary to identify the differently expressed microRNA in DO process. In this study, a comparative miRNA profiling was performed between distraction bones and normal bones. Although lots of time points could be selected in DO process, the end of distraction phase was chosen as the best observation point to analyze the microRNAs expression because distraction phase is the most distinguished period compared to normal fracture healing or bone defect healing process. At this time point, bone regeneration in distraction gap showed obvious with X-ray assays. We speculated that lots of molecular events have been initiated and accumulated during this period. In response to regular tensile force, remarkable change of miRNAs was observed. From the miRNA array data, total 100 miRNAs was found to be differently expressed. Among of which, 74 miRNAs were upregulated and 26 miRNAs were downregulated (Supplementary data Fig. 1). MiR-503 was identified as one of the most differentially expressed miRNAs compared to its expression in normal bone tissue.

As a tumor suppressor, miR-503 was reported to suppress cell proliferation and metastasis in multiple cancers, such as glioma^21^, osteosarcoma^22^, colorectal cancer^23^, breast cancer^24^ and prostate cancer^25^. In recent studies, miR-503 was also reported to regulate bone metabolism. Chen’s study demonstrated that miR-503 could regulate osteoclastogenesis through targeting RANK. Overexpression of miR-503 could prevent bone loss in ovariectomized mice, while silencing of miR-503 could promote bone resorption^26^. Another report indicated miR-503 suppressed proliferation and migration through modulation of FGF2 in osteosarcoma cells^27^. Considering that FGF2 functions as an important positive regulator in osteogenesis, the suppression of FGF2 induced by miR-503 might directly inhibit bone formation. However, our results showed that miR-503 was highly expressed during distraction osteogenesis and overexpression of miR-503 promoted bone formation. As a multi-target regulator, miRNAs could play opposite biological roles in different tissues through different targets.

As we described before, miRNA play critical role in various biological activities through post-transcriptional suppression. By using online bioinformatics tools, Smurf1 was predicted to be a target gene of miR-503 and we also confirmed this prediction by using biological assays. As we all know, Smurf1 is a famous negative regulator in TGFβ/BMP signaling pathway^28, 29^. Our results also indicated osteogenic markers, such as ALP, BMP2 and Runx2, were increased when Smurf1 was silenced by Smurf1 siRNA, a similar effect to miR-503 overexpression. Besides, a rescue effect was observed that miR-503 inhibitor alleviated the enhanced osteogenic effect induced by Smurf1 siRNA. We also found that overexpress smurf1 could reverse the effect of agomir-503 on increasing bone formation (Supplementary Data Fig. 2). Therefore, we confirmed that miR-503 exerted osteogenic functions through suppressing Smurf1 expression, which partially explain the underlying mechanism of DO.

Considering that osteogenesis could be enhanced by miR-503 overexpression, we also investigated the bone formation ability of miR-503 overexpression in animal model of DO *in vivo*. Although numerous methods have been tried to promote bone formation in DO, to our knowledge, it is the first time to apply miRNA therapy to promote bone formation in DO process in animal model. The outcomes demonstrated that bone formation was accelerated in the group with miR-503 overexpression MSCs injection. Therefore, miR-503 overexpression therapy could be used as a novel intervene for promoting bone formation of DO.

Although we demonstrated the osteogenic function of miR-503 *in vitro* and *in vivo*, there were some limitations in this study. For example, we only investigated the relationship between miR-503 expression and bone formation in DO, however, the direct relationship between miR-503 expression and mechanical stimulation has not been fully revealed. Considering that many miRNAs have been reported to be sensitive to the mechanical stimulation, further analysis should lay emphasis on the miR-503 expression when exposed to mechanical stimulation, especially tensile stimulation. On the other hand, we drawn the conclusion according to the data from the cell and DO animal model and it needs to be validated in DO patients.

In summary, our results illustrated that miR-503 was upregulated during distraction phase of DO. Overexpression of miR-503 could promote bone formation *in vitro* and *in vivo* through targeting Smurf1. These findings not only provide potential cues to explain the mechanism of DO in bone formation but also offer preclinical evidences that miR-503 may become a novel therapeutic to promote bone formation in clinic.

## Methods and Materials

### Animals

Three-month old Sprague-Dawley male rats were obtained from the Laboratory Animal Research Centre of The Chinese University of Hong Kong (n = 1 for cell harvest; n = 3 for microarray; n = 10 specimens per group). This study was specifically approved by the Animal Experimentation Ethics Committee of the Chinese University of Hong Kong (AEEC No. 14-052-MIS), and carried out under the animal license issued by the Hong Kong SAR Government. All methods were performed in accordance with the relevant guidelines and regulations. All efforts were made to minimize the number of animals used and their suffering.

### Surgical procedure of DO

All the animals were operated under general anesthesia by ketamine (40 mg/kg) and xylazine (4 mg/kg) given intraperitoneally. Mid-diaphyseal corticotomy was created and a custom made external distraction device was fixed to the tibia by four stainless steel pins. Wounds then were closed in layers and animals were free to move in the cage after the surgery.

### Distraction protocol

After a 5-day latency period, lengthening was initiated at a rate of 0.25 mm/12 hours for 10 days. Then bone segments were maintained the position with external device for another 4 weeks before the animals were sacrificed.

### Sample collection and total RNA extraction

Bone tissues were harvested from distraction gap at the end day of distraction period and immediately thrown into the liquid nitrogen in case of RNA degradation. Then the samples were ground up in RNase-free mortars with liquid nitrogen and TRIzol reagent (Invitrogen, USA) was used to extract the total RNA from the precipitate.

### MicroRNA microarray

An Agilent rat miRNA microarray (8 * 60 K) was used for global scanning of miRNA expression in total RNA samples. Sample labeling, microarray hybridization, and washing were performed based on the manufacturer’s standard protocols (Agilent Technologies Inc., Santa Clara, California, USA). Briefly, total RNA was dephosphorylated, denatured, and then labeled with Cyanine-3-CTP. After purification, labeled RNAs were hybridized onto the microarray. After washing, the arrays were scanned with an Agilent Scanner G2505C (Agilent Technologies Inc., Santa Clara, California, USA). Feature Extraction software (version 10.7.1.1; Agilent Technologies Inc., Santa Clara, California, USA) was used to analyze microarray images and obtain raw data. Next, GeneSpring software (version 12.5; Agilent Technologies Inc., Santa Clara, California, USA) was used to complete the basic analysis using raw data. The raw data was normalized with the quantile algorithm. If the probes with a positive normalized expression value were flagged as “Detected” in at least 100% of samples, they were chosen for further analysis. Differentially expressed miRNAs were then identified through fold change as well as the *p* value calculated using a Student’s *t*-test. The threshold set for significantly up- and down-regulated genes was a fold change >2.0 and a *P* value < 0.05. The miRNA microarray assay was performed by Shanghai OEBiotech Technology Co, Ltd. (Shanghai, China).

### Quantitative Real-Time PCR for osteomarkers

Total RNA from bone tissues or cells extracted with TRIzol Reagent was used to synthesize complementary DNAs (cDNAs) with M-MLV Reverse Transcriptase (Invitrogen) according to the manufacturer’s instructions. Real-time PCR was performed using the Step One Plus Real-Time PCR System (Applied Biosystems, USA). The reaction conditions consisted of 15 µl reaction volumes with diluted cDNA template 3 μL, 7.5 μL SYBR-Green Master Mix (2×), 3.9 μL PCR-Grade water and 0.3 μL of each primer (10 μM). Amplification conditions were as follows: first at 95 °C for 5 min, and then 40 cycles of 95 °C for 15 s and 60 °C for 60 s. Primer sequences were as follows: BMP2 forward: 5′ aaggcaccctttgtatgtgg 3′, reverse: 5′ catgccttagggattttgga 3′; Runx2 forward: 5′ ccgatgggaccgtggtt 3′, reverse: 5′cagcagaggcatttcgtagct 3′; ALP forward: 5′ tccgtgggtcggattcct 3′, 5′ gccggcccaagagagaa 3′. The relative quantification of gene expression was analyzed with 2^−ΔΔCT^ method, normalized with GAPDH expression level.

### Quantitative Real-Time PCR for miR-503 expression level

Total RNA was extracted with TRIzol (Invitrogen) and then reverse transcribed into cDNA using M-MLV Reverse Transcriptase (Invitrogen) and miRNAs were collected with All-in-One miRNA quantitative reverse transcription- (qRT-) PCR detection kit (GeneCopoeia, Guangzhou, China) according to the manufacturer’s instructions. Real-time PCR was performed using the Step One Plus Real-Time PCR System (Applied Biosystems, USA), as indicated in the instructions. To analyze the expression level of miR-503-5p, a total reaction volume of 20 *μ*L contained 10 *μ*L SYBR Mix, 5.6 *μ*L RNase-free water, 1 *μ*L miR-503-5p primer, 1 *μ*L universal adaptor PCR primer, 2 *μ*L cDNA template, and 0.4 *μ*L ROX. Amplification and detection were performed as follows: 95 °C for 10 min and then 40 cycles of 95 °C for 15 s, 60 °C for 30 s, and 72 °C for 20 s. Primer sequences were as follows: mir-503-5p 5′ tagcagcgggaacagtactgcag 3′; U6 forward 5′ ctcgcttcggcagcaca 3′, reverse 5′ aacgcttcacgaatttgcgt 3′. The relative quantification of gene expression was analyzed with 2^−ΔΔCT^ method, normalized with U6 expression level.

### Cell culture

The rBMSCs were isolated as previously described. Briefly, the rBMSCs were obtained from the bone marrow of 4-week old SD rat and cultured in a 100 mm cell culture dish in the alpha complete culture medium at 37 °C with 5% CO_2_ and 95% humidity. The rBMSCs from passages 3–5 were used in the experiments. The surface antigens of rBMSCs were detected by flow cytometry using CD90, CD44, CD34 and CD45 (data not shown).

### Transfection of mimic/inhibitor

The miR-503 mimics and inhibitor were obtained from Genepharma Company (Genepharma, China). The transfection of agomir-503 or antagomir-503 were performed with Lipofectamine 2000 (Invitrogen, USA) according to the manufacturer’s instruments.

### Osteogenic differentiation and ALP/Alizarin Red S staining

Briefly, the medium was removed and replaced by osteogenic induction medium (1 nM dexamethasone, 50 mM L-ascorbic acid-2-phosphate and 20 mM β-glycerolphosphate with complete medium). The induction medium was changed every 3 days. 3 days and 14 days after the induction, ALP and Alizarin Red S staining were performed separately to evaluated positive rate of alkaline phosphatase and calcium deposit formation.

### Western blot analysis

Cells were washed by cold PBS twice and lysed by extraction buffer (Invitrogen, USA). Protein fractions were collected by centrifugation at 15,000 g at 4 °C for 10 min. Equal proteins were loaded onto 10% Tris/glycine gels for electrophoresis and then transferred to a PVDF membrane (Millipore, Bedford, MA) and blocked in 5% non-fat milk (Biorad, USA) for 1 h at room temperature with rocking. Then, the primary antibody, anti-Smurf1 (1:100, Santa Cruz, USA) and anti-GAPDH (1:100, Santa Cruz, USA) was added and incubated at 4 °C overnight. After washing in TBST for three times (5 min for each time), the membrane was incubated with horseradish peroxidase-linked secondary antibodies (anti-mouse or anti-goat) for 1 h at room temperature. Following three TBST washes, protein was detected with the enhanced chemiluminescence (ECL) blotting reagents (Amersham Biosciences, USA) according to the manufacturer’s instruction.

### Smurf1 3′ UTR cloning and luciferase Assay

A fragment of the Smurf1 3′UTR containing the predicted binding site or their mutant fragment sequence on each side with suitable enzyme cleavage sites were synthesized and cloned into downstream of the Luciferase reporter gene (Smurf1 Wt-Luc or Smurf1 Mu-Luc). Each vector, along with Relina vector and miR-503 mimics or negative control, were transfected into 293 T cells using Lipofectamine 2000 reagent (Invitrogen, USA) following the instructions. Cells were harvested 48 h after transfection and luciferase activity was detected using the Dual-Luciferase Reporter Assay System (Promega, USA).

### Overexpression of miR-503 in rBMSCs

To generate pLL3.7-pre-miR-503, the oligonucleotides encoding pre-miR-503 were amplified and cloned into the XhoI site of pLL3.7 under the control of U6 promoter. Scrambled control plasmid was also constructed according to the method used by Splinter *et al*.^30^. The pseudolentiviruses were produced by transfection of 293FT packaging cells (Invitrogen, USA) using the calcium phosphate method. For transduction, 1 × 10^5^ cells were seeded into 6-well plate and incubated with lentiviruses and 8 *μ*g/mL polybrene in the incubator for 24 h^31^.

### Local injection therapy

Mesenchymal stem cells modified by microRNAs intervention were local injected into the distraction gap percutaneously monitored by X-ray. All the injections were given on the first day of consolidation. At the end of experiment, all animals were sacrificed and the tibial specimens were harvested for further analysis.

### Microcomputer Tomography (Micro-CT) Examination

MicroCT analysis was performed for each animal after sacrificed. Briefly, all the specimens were imaged using a vivaCT 40 (Scanco Medical) with a voltage of 70 keV, a current of 114 *μ*A, and 10.5 *μ*m isotropic resolution. To eliminate the interference by the native bone, the central 150 layers in horizontal plane of the distraction bone were selected as the region of interest. Low- and high-density mineralized tissues were reconstructed using different thresholds (low attenuation = 158, high attenuation = 211) using our established evaluation protocol with small modification. The high-density tissues (threshold between 211 and 1000) represented the newly formed highly mineralized calluses and the original cortices, while the low-density tissues (threshold between 158 and 211) represented the newly formed calluses. The threshold between 158 and 1000 represents the whole bone tissues. Bone volume (BV), tissue volume (TV), and BV/TV (bone volume fraction) of each sample were recorded for analysis.

### Four-Point Bending Mechanical Testing

Mechanical test was performed within 24 hours after sacrificed under room temperature. A four-point bending device (H25KS; Hounsfield Test Equipment Ltd. UK) with a 200 N load cell was used to test the distracted tibiae to failure. The tibiae were loaded in the anterior-posterior direction with the inner and outer span of the blades set as 8 and 20 mm, respectively. The long axis of the tibia was oriented perpendicular to the blades during the test. The ultimate load, the energy to failure, and the modulus of elasticity (E-modulus) were recorded and analyzed using built-in software (QMAT Professional; Tinius Olsen, Inc., Horsham, PA, USA).

### Histological Analysis

The femora were fixed in 10% buffered formalin, decalcified with 9% formic acid, and embedded in paraffin. Attempts were made to standardize the sectioning at a midsagittal plane of each specimen by cutting the specimen in half (longitudinally in a sagittal plane) using a slicing blade. Thin sections (5 *μ*m) are cut by a Rotary Microtome (HM 355 S, Thermo Fisher Scientific, Inc., Germany) along the long axis of each femur in sagittal plane. Hematoxylin and eosin (HE) staining was performed using standard protocols after deparaffinization.

### Bone histomorphometric analysis

After sacrificed, the distracted tibiae were dehydrated in graded concentrations of ethanol and embedded into methyl methacrylate (MMA) with our previously established protocol after microCT analysis. Sagittal sections of distracted tibia in 5 μm was performed with a Leica SM2500E microtome (Leica Microsystems, Germany). The sections were then subjected to Goldner’s trichrome staining to analyze bone dynamic histomorphometric parameters, including MAR and MS/BS with fluorescence microscopy (Leica image analysis system, Q500MC) and OsteoMeasure system (OsteoMetrics Inc., Decatur, GA, USA). The bone histomorphometric parameters were calculated and expressed according to the standardized nomenclature for bone histomorphometry.

### Statistical Analysis

All quantitative data were transferred to statistical spreadsheets and analyzed by a commercially available statistical program SPSS version 16.0 (IBM, USA); independent *t*-test was used for comparison of mean values with *P* < 0.05 considered as statistically significant.

## Electronic supplementary material

Supplementary data
