# Supplementary material for: MiR-503 Promotes Bone Formation in Distraction Osteogenesis through Suppressing Smurf1 Expression
Source: Sci Rep. 2017 Mar 24;7:409. doi: 10.1038/s41598-017-00466-4 (PMC5428455; doi:10.1038/s41598-017-00466-4)
Supplement: Supplementary file 1 — Supplementary data [file 41598_2017_466_MOESM1_ESM.pdf]

MiR-503 Promotes Bone Formation in Distraction Osteogenesis through Suppressing Smurf1 Expression

Yuxin SUN[1,2,3#], Jia XU[4#], Liangliang XU[2,3], Jinfang ZHANG[2,3], Kaiming CHAN[2], Xiaohua PAN[1] *, Gang LI[1,2,3,5] *

1. *Department of Orthopaedics and Traumatology, Bao-An District People's Hospital, Shenzhen, PR China.*
2. *Department of Orthopaedics & Traumatology, Li Ka Shing Institute of Health Sciences and Lui Che Woo Institute of Innovative Medicine, Faculty of Medicine, The Chinese University of Hong Kong, Prince of Wales Hospital, Shatin, Hong Kong SAR, PR China.*
3. *The CUHK-ACC Space Medicine Centre on Health Maintenance of Musculoskeletal System, The Chinese University of Hong Kong Shenzhen Research Institute, Shenzhen, PR China.*
4. *Department of Orthopaedic Surgery, Shanghai Jiaotong University Affiliated Sixth People's Hospital, Shanghai, PR China.*
5. *Key Laboratory for Regenerative Medicine, Ministry of Education, School of Biomedical Sciences, Faculty of Medicine, The Chinese University of Hong Kong, Hong Kong SAR, PR China.*

#Yuxin Sun and Jia Xu contributed equally to this work.

*Correspondence author at: Room 904, Li Ka Shing Institute of Health Sciences, The Chinese University of Hong Kong, Prince of Wales Hospital, Shatin, NT, Hong Kong, SAR, PR China.

Tel: (852) 3763 6153; Fax: (852) 2646 3020; E-mail: gangli@cuhk.edu.hk

Supplementary data

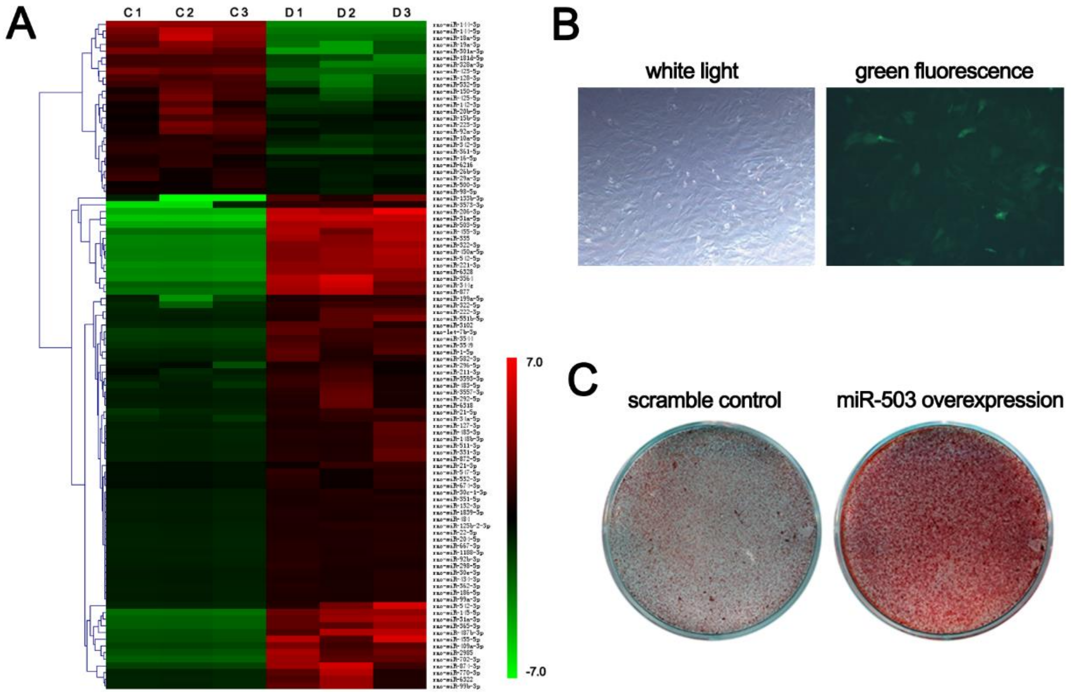

S1. (A) As shown in heat map of microarray data, total 100 miRNAs were found differentially expressed, with 74 miRNAs upregulated and 26 miRNAs downregulated. C represents control group and D represents DO group, red represents up regulation while green represents down regulation. (B) Pictures demonstrated the transfection efficiency of miR-503 overexpression lentivirus. The pLL3.7-pre-miR-503 was labeled by GFP. The rBMSCs would show fluorescent green after successfully transfection. According to the results, the transfection rates would be 70% - 80%. (Magnification=50×) (C) More calcium deposits were found in miR-503 overexpression group than in scramble group in alizarin red staining after transfected by the lentivirus.

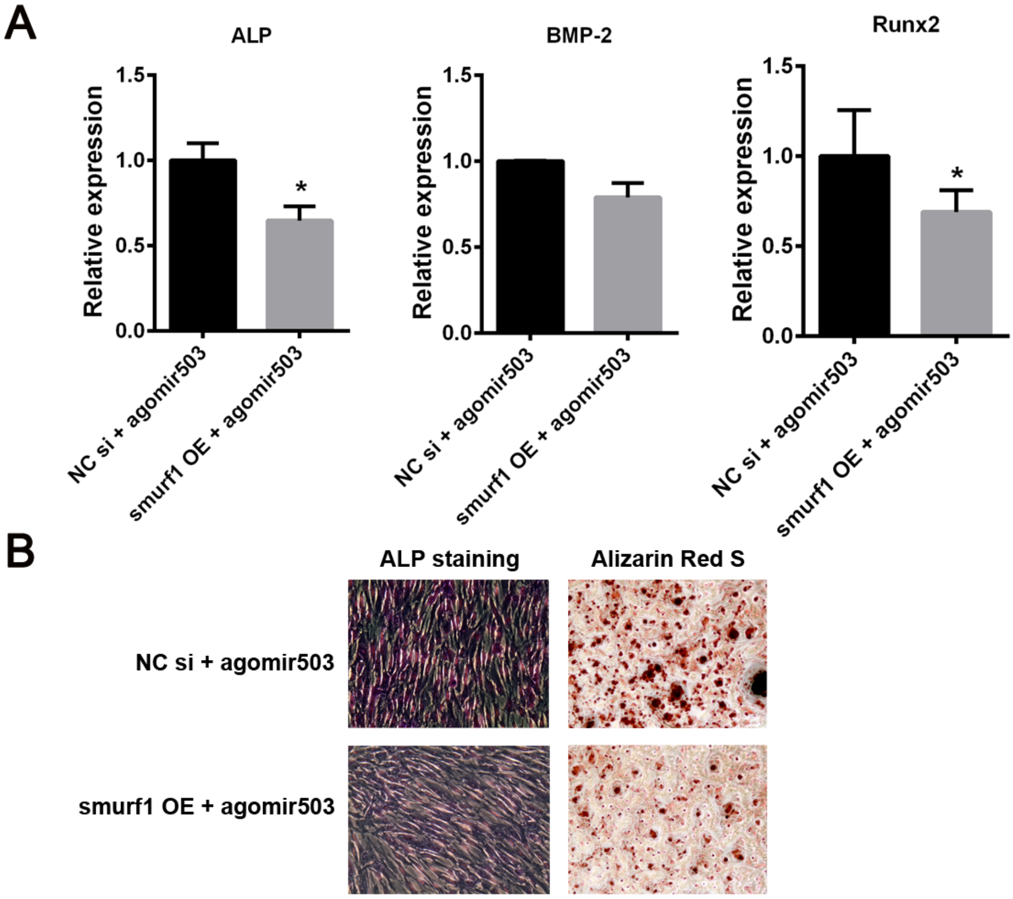

S2. **Overexpression of Smurf1 would decrease the osteogenic effect of miR-503 elevation.** (A) As shown in the picture, expression level of ALP, BMP2 and Runx2 were performed in two groups. In treatment group, smurf1 overexpression plasmid and agomir-503 were co-transfected into the BMSCs. In control group, blank control plasmid and agomir-503 were co-transfected into the BMSCs. Lipofectamine 2000 was used as the transfection agent. The expression level of ALP and Runx2 were found significant lower in treatment group than that in control group. (B). Three days after the transfection, osteogenic differentiation was performed by osteogenic induction medium (OIM). ALP staining was performed 3 days after the osteogenic differentiation and Alizarin red S was performed 10 days after the osteogenic differentiation. Results showed that the number of ALP positive cells and calcium nodules were decreased after the co-transfection of smurf1 overexpression plasmid and agomir-503. (ALP staining magnification=20×, Alizarin red S magnification=10×) (Smurf1 overexpression plasmid information: Name: PCMV5B-Flag-Smurf1 wt; Addgene Code: 11752)

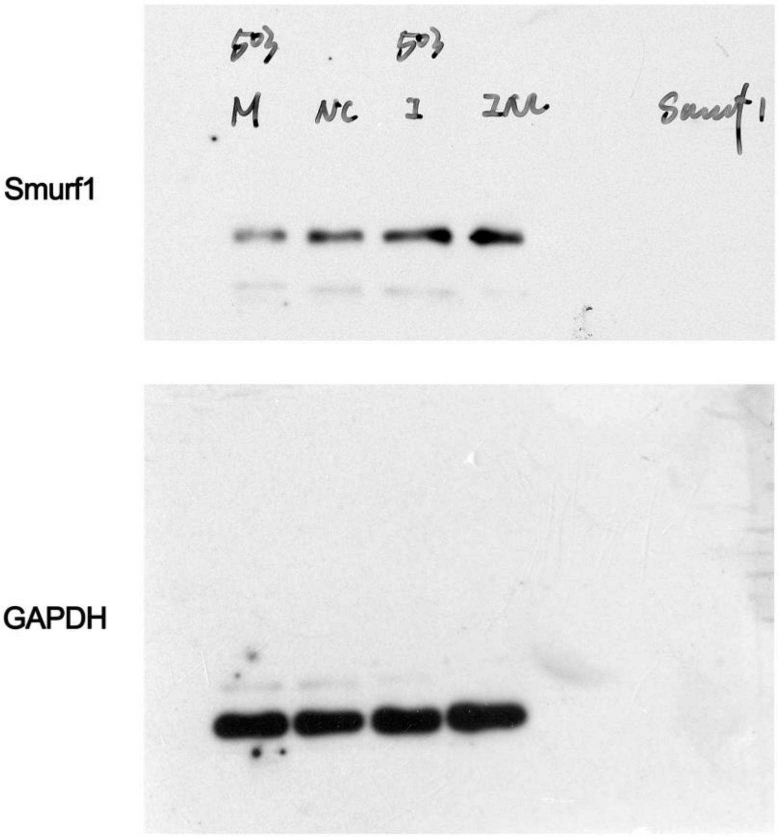

S3. Uncropped image of the western blot result. As shown in the picture, the blots above represent the expression of smurf1 protein. After agomir-503 transfected, expression of smurf1 were decreased. Conversely, transfection antiagomir-503 would increase the expression of smurf1. (GAPDH was used as loading control)
